# The utility of computed tomography-derived inferior vena cava parameters in predicting outcomes in patients with active bleeding undergoing transarterial embolization

**DOI:** 10.1186/s12245-025-01033-9

**Published:** 2025-10-20

**Authors:** Hans-Jonas Meyer, Veronika Sotikova, Simon Riegelbauer, Sebastian Ebel, Holger Gößmann, Matthias Mehdorn, Uwe Scheuermann, Hans-Michael Tautenhahn, Christian Kleber, Timm Denecke, Manuel F. Struck

**Affiliations:** 1 Department of Diagnostic and Interventional Radiology, University Hospital Leipzig, Liebigstr. 20, 04103 Leipzig, Germany; 2 Department of Visceral, Transplant, Thoracic and Vascular Surgery, University Hospital Leipzig, University of Leipzig, Liebigstr. 20, 04103 Leipzig, Germany; 3 Department of Orthopedics, Trauma and Plastic Surgery, University Hospital Leipzig, Liebigstr 20, 04103 Leipzig, Germany; 4 Department of Anesthesiology and Intensive Care Medicine, University Hospital Leipzig, Liebigstr. 20, 04103 Leipzig, Germany

**Keywords:** Inferior vena cava volume, Inferior vena cava diameter, CT, Active bleeding, Transarterial embolization, Massive transfusion, Mortality

## Abstract

**Background:**

The inferior vena cava (IVC) parameters are associated with prognostic significance in emergency patients, but there is a lack of data using this parameter in patients with active bleeding.

**Objectives:**

To investigate the prognostic relevance of IVC parameters in patients with active bleeding.

**Patients and methods:**

A retrospective analysis was conducted on consecutive patients who underwent transarterial embolization due to bleeding from different anatomical sites following computed tomography (CT) imaging at a university medical center over a five-year period (2018–2022). The initial CT scan was used to determine the IVC volume and IVC flatness index, which were then incorporated into multivariable regression analyses that included demographic, hemodynamic, and laboratory data.

**Results:**

The analysis included 188 patients (75.3% male) with a median age of 50 years, and a massive transfusion rate and an all-cause 30-day mortality rate of 26.6% each. Compared with female patients, male patients had a significantly higher median IVC volume (25.45 vs. 15.8 cm³, *p* < 0.001), whereas the median IVC flatness index was similar for both sexes (14 vs. 14, *p* = 0.414). Median IVC volumes were similar between 30-day survivors and nonsurvivors (21.6 vs. 20.2 cm³, *p* = 0.382) and between patients who underwent massive transfusion and those who did not (21.2 vs. 21.5 cm³, *p* = 0.567). A multivariable Cox proportional hazards model revealed a statistically significant association between the IVC flatness index and 30-day mortality (hazard ratio, 1.27; 95% confidence interval, 1.01–1.59; *p* = 0.038). Additionally, logistic regression analysis revealed no significant association between the IVC flatness index and massive transfusion (univariable odds ratio, 1.01; 95% confidence interval, 0.75–1.34; *p* = 0.972).

**Conclusions:**

A higher IVC flatness index was associated with 30-day mortality in patients undergoing transarterial embolization for active bleeding. Further studies are needed to determine the prognostic value of CT-derived IVC parameters.

**Supplementary Information:**

The online version contains supplementary material available at 10.1186/s12245-025-01033-9.

## Introduction

The utilization of multi-slice computed tomography (CT) scans has become a standard diagnostic procedure for patients with acute bleeding. This imaging modality plays a crucial role in localizing the bleeding site and formulating a comprehensive treatment plan for endovascular interventions [1, 2].

A growing trend involves the use of CT images not only for diagnostic purposes, but also to define image-based prognostic parameters and scores [3–5]. This facilitates more comprehensive patient assessment and improved prediction of disease outcomes. Given that the vast majority of patients with acute bleeding undergo emergent CT scans, it is important to extract prognostic factors from these images.

The inferior vena cava (IVC) volume on CT has been identified as a prognostic imaging marker in critically ill patients, as a surrogate parameter of the actual volume status and cardiac preload [6]. The majority of studies examining IVC volume in acute trauma patients have employed the IVC flatness index, that is calculated by dividing the transverse diameter by the anteroposterior diameter [7, 8]. These have been associated with short-term mortality [9–11], occult shock [7, 12], and massive transfusion [13, 14]. However, the utilization of these IVC features has also been associated with a low prognostic capability [15, 16], and there remains a paucity of data concerning critically ill non-trauma patients.

Modern CT imaging analysis tools can now perform volumetry in a matter of seconds. This eliminates the need for other measurements, such as the index/diameter of the IVC in a single axial slice. However, there is currently no evidence whether IVC volumetry or IVC flatness index is a better outcome predictor in patients with active bleeding. The primary objective of the present study was to identify associations between inferior vena cava (IVC) parameters as measured in the CT scans, and all-cause 30-day mortality. The secondary objective was to identify associations with massive transfusion.

## Methods

A retrospective analysis was conducted on consecutive patients who underwent transarterial embolization for acute bleeding at a tertiary referral center between 2018 and 2022. The study was conducted subsequent to the approval of the local ethics committee (ethical code 319/24-ek), in accordance with the ethical standards established by the institutional and/or national research committee, as well as the Helsinki Declaration of 1964 and its subsequent amendments.

**Fig. 1:**
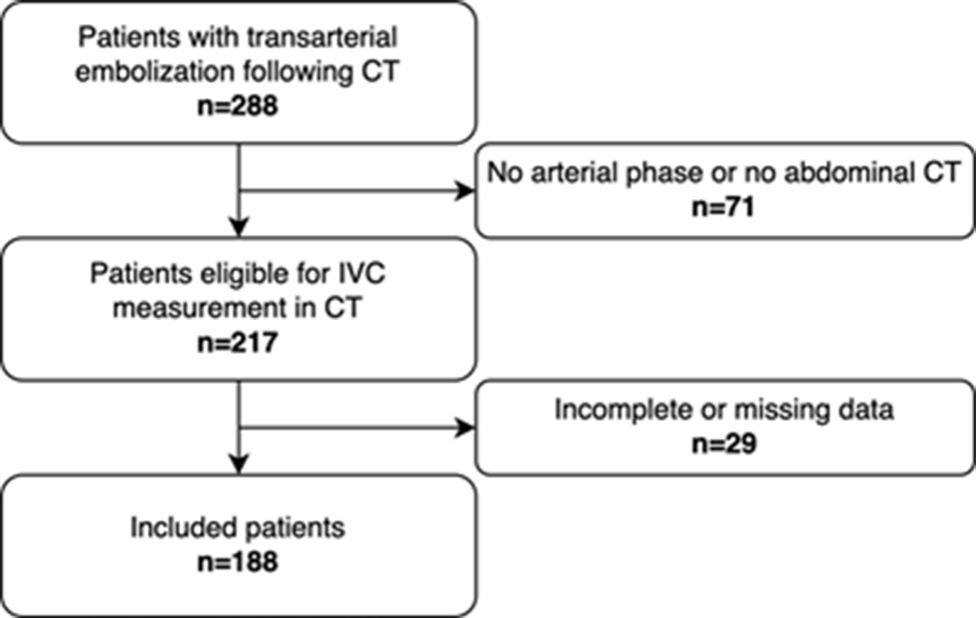
Study flow chart of the present study

The inclusion criteria were as follows: (1) CT images that were obtained within 24 h before the angiography procedure, including a CT scan of the abdomen for the VCI measurement, (2) availability of data regarding blood transfusion, massive transfusion, and all-cause 30-day mortality, (3) availability of demographic data, body size metrics, hemodynamic data, and laboratory data. Figure 1 shows the study flow chart.

Analysis of patient’s characteristics included sex, age, body height, body weight, body mass index (BMI), systolic blood pressure, diastolic blood pressure, heart rate, shock index, diastolic shock index, hemoglobin level, platelet count, transfusion of blood products, number of transfused packed red blood cell (PRBC) units, massive transfusion (defined as ≥ 10 units of PRBC within 24 h or ≥ 5 units of PRBC within 4 h), and 30-day mortality. Localization of bleeding was stratified by bleeding cause and body region. The procedural time and technical success of the angiographic intervention were recorded.

Patients were excluded from the study if imaging or clinical data were not obtained within the specified time frame or if CT imaging was performed without the administration of contrast media. Acute bleeding was defined as exhibiting clinical symptoms of bleeding, accompanied by a decrease in hemoglobin levels, hemorrhagic shock, and the detection of bleeding through endoscopy.

### Transarterial embolization

The decision for transarterial embolization was made by a multidisciplinary team based on clinical symptoms, evidence of blood extravasation on endoscopy, contrast-enhanced CT or digital subtraction angiography (DSA), and laboratory values. TAE was performed by board-certified interventional radiologists with a minimum of five years of experience in interventional radiology. Access was typically performed via a transfemoral approach and rarely via a transbrachial approach. The embolic agent was selected based on vascular anatomy, local hemodynamics, microcatheter stability, mechanism of bleeding, and the experience of the interventionalist. The different materials and devices used for TAE included embolization particles (Contour PVA; Boston Scientific), microspheres (Embozene; Boston Scientific), sponges (Gelaspon; Bausch + Lomb), n-butyl-2-cyanoacrylate adhesives (Histoacryl; B. Braun), liquid embolic systems (Onyx; Covidien), and 0.018” microcoils (Hilal or Tornado (SEF and LEF); Cook Medical). Vessel plugs (Amplatzer; St. Jude Medical) were also used. Depending on the clinical situation and the interventionalist´s preference, the embolic materials were used either alone or in combination.

### Imaging technique

Contrast-enhanced, triphasic (native, arterial and portal-venous) CT was performed in a clinical setting using a 128-slice CT scanner (Ingenuity 128, Philips, Hamburg, Germany). In each case, the indication for the CT was to locate the source of bleeding. Intravenous iodinated contrast agent (90 mL Imeron 400 MCT, Bracco Imaging Germany GmbH, Konstanz, Germany) was administered at a rate of 4.0 mL/s via a peripheral venous line. Automatic bolus tracking was performed in the descending aorta with a trigger of 100 Hounsfield units (HU). Typical imaging parameters were: 100 kVp; 125 mAs; slice thickness, 1 mm; pitch, 0.9.

### IVC volume measurement and IVC flatness index calculation

**Fig. 2:**
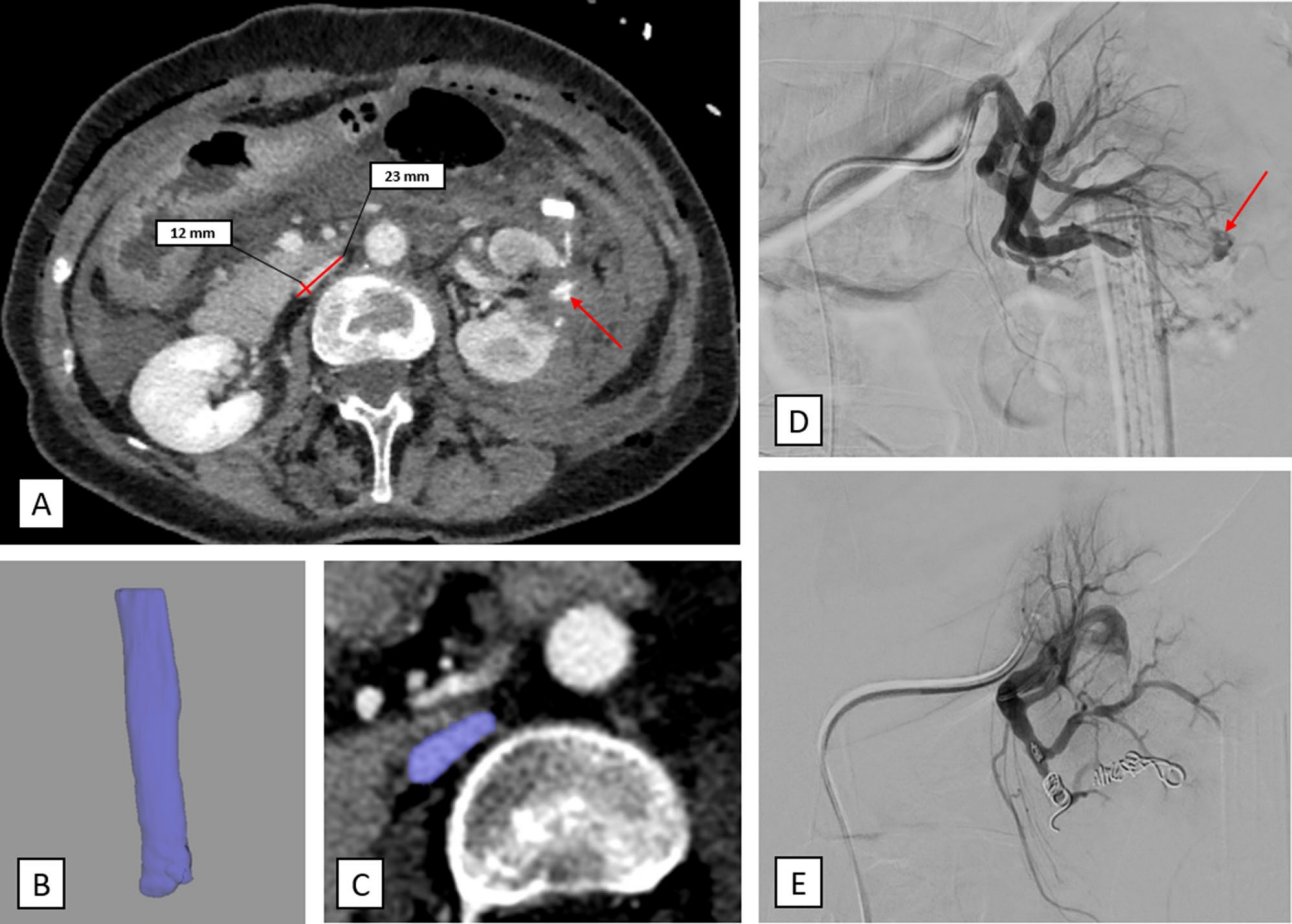
Representative case example of the cohort. **A** 62-years old female patient with postoperative active bleeding of the left kidney. The red arrow demonstrates the contrast media extravasation of the venous phase image. The flat IVC is measured with a long axis diameter of 23 mm and a short axis diameter of 12 mm with a flatness index of 1.9. **B** and **C** Volumetry of the IVC with a volume of 9.2 cm³. **D** Angiography with the catheter in the left renal artery. The red arrow demonstrates the contrast media extravasation. **E.** The bleeding stopped after coil embolization

The IVC volume was measured semiautomatically by a trained radiologist using the volumetry tool of the Philips advanced visualization workspace (version 15, Philips Healthcare, Hamburg, Germany) on the venous phase images. The measurement was taken distally from the confluence of the iliac veins to the level of the diaphragm using the self-expanding volume tool, as employed in previous studies [14, 17]. The IVC flatness index was defined as the transverse-to-anteroposterior diameter ratio at the level below the renal veins, which is comparable to previous research [7]. The reader was blinded to the patient’s clinical characteristics and outcomes. Figure 2 provides a measurement of a representative patient of the cohort.

### Statistical analysis

The categorical variables of the dataset were presented by absolute numbers and percentages, while the continuous variables were described by medians and interquartile ranges (IQR, quartile 1 and quartile 3). Subsequent to the testing for normal distribution via the Kolmogorov-Smirnov test, group differences were calculated using the Mann-Whitney U test, Student’s t-test, and Chi-square test, when applicable. The relationship between IVC volume and the other variables was investigated using Spearman´s correlation coefficient. The association between the variables and 30-day mortality was analyzed using the Cox proportional hazard model. To identify independent predictors of massive transfusion, multivariable logistic regression analysis was conducted. The results of the univariable analyses, which identified statistically significant predictors, were included in the multivariable models. Variables were chosen to provide at least ten events per variable to account for type I errors. The hazard ratios (HR) for 30-day mortality and odds ratios (OR) for massive transfusion, were provided including 95% confidence intervals (CI). In all instances, p-values < 0.05 were considered statistically significant. The statistical analysis was performed using R 4.2.2 (R Foundation for Statistical Computing, Vienna, Austria), DATAtab (DATAtab e.U., Graz, Austria) and GraphPad Prism version 10.0.2 for MacOS (GraphPad Software, Boston, Massachusetts, USA).

## Results

The study cohort consisted of 188 patients (124 males; 65.9%) with a median age of 66.7 years (Table 1). The primary causes of bleeding were: postoperative/post-interventional (*n* = 54, 28.7%); trauma-related (*n* = 26, 13.8%); pseudoaneurysm (*n* = 19, 10.1%); tumor-associated (*n* = 16, 8.5%); ulcer (*n* = 14, 7.4%); and other causes (*n* = 56, 29.8%). The anatomical bleeding sites were the upper gastrointestinal tract (*n* = 34, 18.1%), the thoraco-lumbar wall (*n* = 33, 17.6%), the lower gastrointestinal tract (*n* = 32, 17%), the liver (*n* = 26, 13.8%), kidney (*n* = 23, 12.2%), pelvis (*n* = 18, 9.6%), spleen (*n* = 14, 7.4%), lower extremity artery (*n* = 4, 2.1%), pancreas (*n* = 3, 1.6%), and pulmonary artery (*n* = 1, 0.5%). The median (IQR) procedural time was 56 min (range, 37.8 to 84.3 min), with minimum times of seven minutes and maximum times of 192 min. Technical success was achieved in 179 patients (95.2%). Of the nine unsuccessful cases, five survived, and three received massive transfusions.

**Table 1:** Characteristics of the study cohort related to 30-day mortality

Parameter *n* (%), median (IQR)	All patients (*n* = 188; 100%)	Survivors (*n* = 138; 73.4%)	Non-survivors (*n* = 50; 26.6%)	*p*-value
Sex, male	124 (65.9)	93 (67.4)	31 (62)	0.491
Age, years;	66.7 (31; 64)	65.9 (31; 63)	71.4 (31; 70)	**0.043**
Height, m	1.72 (1.65; 1.8)	1.73 (1.66; 1.80)	1.71 (1.65; 178)	0.371
Weight, kg;	77 (65; 90)	75 (65; 90)	80 (70; 89.75)	0.395
BMI, kg/m^2^;	25.7 (22.9; 29.2)	24.9 (22.6; 29.1)	26.6 (23.8; 29.4)	0.181
IVC vol., cm^3^;	21.6 (15.8; 30.9)	21.1 (16; 31.2)	20.2 (13.4; 28.6)	0.382
IVC t. diameter, cm;	26 (23; 29)	26 (23; 29)	26 (23; 29)	0.747
IVC a.p. diameter, cm	14 (8; 17)	14 (9; 18)	11.5 (7; 16)	**0.025**
IVC, flatness index;	1.93 (1.55; 2.88)	1.86 (1.5; 2.68)	2.27 (1.75; 3.28)	**0.016**
SBP, mmHg	110 (92; 135)	116.5 (99.25; 137.75)	100 (86.25; 121.5)	**0.003**
DBP, mmHg	63 (50;79)	65 (55.25; 80)	54.5 (42.25; 70)	**0.003**
Heart rate, bpm	85 (75; 103)	84 (73.25; 100)	94.5 (78; 107)	**0.049**
SI, heart rate/SBP	0.74 (0.61; 1.0)	0.71 (0.56; 0.93)	0.86 (0.71; 1.1)	**0.001**
SI positive	47 (25)	28 (20.3)	19 (38)	**0.013**
DSI, heart rate/DSI	1.33 (1.1; 1.76)	1.23 (1.05; 1.59)	1.57 (0.57; 2.57)	**< 0.001**
DSI positive	153 (81.4)	110 (79.7)	43 (86)	0.321
Hb level	4.9 (4.1; 6)	5.1 (4.2; 6.1)	4.35 (3.9; 5.7)	**0.016**
PLT level, GPT	203 (138; 301.5)	208 (150; 323.25)	189 (118; 270)	0.193
Any pRBC transfusion	123 (65.4)	87 (63)	36 (72)	0.254
pRBC, n;	2 (0; 6)	2 (0; 5)	4.5 (3.9; 5.7)	**0.01**
Massive transfusion	50 (26.6)	31 (22.5)	19 (38)	**0.033**
Procedural time, min	56 (37.8; 84.3)	57 (38; 75)	51.5 (33.3; 68.5)	0.152
Technical success	179 (95.3)	133 (96.4)	46 (92)	0.392

IQR, interquartile range; BMI, body mass index; IVC, inferior vena cava; vol., volume; t.d., transverse diameter; a.p.; anterior-posterior diameter; f.i., flatness index (a.p./t.d.); SBP, systolic blood pressure; DBP, diastolic blood pressure; SI, shock index; DSI, diastolic shock index; Hb, hemoglobin; PLT, platelet count; pRBC, red blood cell unit; bold numbers indicate statistical significance (*p* < 0.05)

PRBC transfusion within the first 24 h was performed in 123 patients (65.4%), including 50 (26.6%) who received massive transfusion. The all-cause 30-day mortality was 26.6% (50 patients).

### Inferior Vena Cava measurement

The IVC volume, transverse diameter, anterior-posterior diameter, and flatness index were assessed in all patients and compared in terms of massive transfusion, 30-day survival, and sex (Table 2). The median anthropometric variables of body height, body weight, and IVC volume were significantly lower in female patients. However, indices of BMI and IVC flatness were comparable between sexes (Table 2). Both IVC volume and the IVC flatness index correlated with diastolic blood pressure (*r* = 0.15 and *r* = -0.17, respectively). Additionally, IVC volume correlated with heart rate, shock index, diastolic shock index, body height, body weight, and BMI (Table 3).

**Table 2:** Anthropometric measurements comparing male and female patients of the study cohort

Parameter Median (IQR)	Male (*n* = 124)	Female (*n* = 64)	*p*-value
Height, m	1.76 (1.71; 1.81)	1.64 (1.58; 1.68)	**< 0.001**
Weight, kg	80 (70; 90)	70 (56.5; 84.25)	**< 0.001**
BMI, kg/m^2^	25.66 (23.02; 28.73)	25.76 (22.04; 29.4)	0.958
IVC volume, cm^3^	25.45 (17.55; 32.58)	15.8 (12.1; 25.1)	**< 0.001**
IVC transverse diameter, cm	26 (23.75; 29)	26 (22.75; 29)	0.405
IVC anteroposterior diameter, cm	14 (9; 17)	14 (7.75; 17)	0.414
IVC flatness index	1.93 (1.57; 2.72)	1.92 (1.49; 3.17)	0.723

BMI, body mass index; IVC, inferior vena cava; bold numbers indicate statistical significance (*p* < 0.05)

**Table 3:** Correlation analyses of associations with inferior Vena Cava volume and flatness index

Parameter	IVC volume *r*	*p*-value	IVC flatness index *r*	*p*-value
Age	-0.14	0.054	0.05	0.455
Height	0.35	**< 0.001**	-0.04	0.58
Weight	0.41	**< 0.001**	-0.09	0.23
BMI	0.3	**< 0.001**	-0.04	0.561
SBP	0.07	0.323	-0.13	0.07
DBP	0.15	**0.04**	-0.17	**0.018**
Heart rate	-0.18	**0.014**	-0.03	0.724
SI	-0.18	**0.018**	0.08	0.269
DSI	-0.24	**0.001**	0.11	0.146
Hb level	-0.05	0.547	0.03	0.699
PLT count	-0.09	0.216	0.13	0.082
pRBC, n	-0.03	0.728	0.06	0.419
Procedural time	0.1	0.164	-0.04	0.586

BMI, body mass index; SBP, systolic blood pressure; DBP, diastolic blood pressure; DSI, diastolic shock index (HR/DBP); Hb, hemoglobin; PLT, platelet count; pRBC, packed red blood cell; bold numbers indicate statistical significance (*p* < 0.05). All correlations using Spearman´s correlation coefficient

### 30-day mortality

Direct comparisons of survivors and non-survivors revealed that non-survivors were significantly older, had a lower IVC anterior-posterior diameter and a higher IVC flatness index. They also had lower median systolic and diastolic blood pressure, a higher median heart rate, a higher median systolic and diastolic shock index, a lower median hemoglobin level, a higher median PRBC amount, and a higher rate of massive transfusion. In univariable Cox proportional hazard analysis, age, IVC anterior-posterior diameter, and PRBC amount were not statistically significant (Table 4). The multivariable model included the three most significant univariable predictors of radiological, hemodynamic, and laboratory variables, with 16.6 events per variable for each. Thus, the IVC flatness index (HR 1.27, 95% CI 1.01–1.59, *p* = 0.038) remained statistically significant after adjusting for the shock index (HR 4.44, 95% CI 1.96–10.05, *p* < 0.001), and hemoglobin level (HR 0.71, 95% CI 0.56–0.9, *p* = 0.005) (model ROC AUC 0.704). When the model included only non-trauma patients (*n* = 161), the IVC flatness index was confirmed to be an independent association (HR 1.36, 95% CI 1.10 − 1.75, *p* = 0.006) when adjusted for the shock index (HR 4.95, 95% CI 2.11–11.65, *p* < 0.001) (Supplemental Table 1).

**Table 4:** Cox proportional hazard models of associations with 30-day mortality

Parameter	Univariable HR	95% CI	*p*-value	Multivariable HR	95% CI	*p*-value
Age	1.02	1–1.04	0.05			
IVC AP diameter	0.96	0.91–1	0.075			
IVC flatness index	1.34	1.08–1.65	**0.007**	1.27	1.01–1.59	**0.038**
SBP	0.99	0.98–1	**0.012**			
DBP	0.98	0.96–0.99	**0.006**			
Heart rate	1.01	1–1.03	**0.025**			
SI	4.3	1.98–9.34	**< 0.001**	4.44	1.96–10.05	**< 0.001**
DSI	1.6	1.26–2.01	**< 0.001**			
Hb level	0.75	0.59–0.95	**0.018**	0.71	0.56–0.9	**0.005**
pRBC (n)	1.01	1–1.04	0.183			
Massive transfusion	1.86	1.05–3.29	**0.033**			

HR, hazard ratio; CI, confidence interval; IVC, inferior vena cava; AP, anterior-posterior diameter; SBP, systolic blood pressure; DBP, diastolic blood pressure; SI, shock index; DSI, diastolic shock index (HR/DBP); Hb, hemoglobin; pRBC, packed red blood cell units; bold numbers indicate statistical significance (*p* < 0.05). Univariable analysis including statistically significant variables of comparisons of survivors and non-survivors (Table 1). Multivariable analysis including the three most significant univariable predictors of IVC parameters, hemodynamic variables, and laboratory variables (providing 16.6 events per variable)

### Massive transfusion

The median IVC volumes of patients who underwent massive transfusion were similar to those who did not (21.2 vs. 21.55 cm^3^, *p* = 0.657). Univariable logistic regression analysis of massive transfusion revealed statistically significant associations with body weight, BMI, diastolic blood pressure, hemoglobin level, and platelet count. Age, sex, height, IVC volume, IVC transverse diameter, IVC anterior-posterior diameter, IVC flatness index, systolic blood pressure, heart rate, shock index and diastolic shock index were not significantly associated (Table 5). Multivariable analysis, which included the three most significant predictors of body size, hemodynamic variables, and laboratory variables, revealed body weight (OR 1.03, 95% CI 1.01–1.05, *p* = 0.004) and hemoglobin level (OR 0.54, 95% CI 0.39–0.74, *p* < 0.001) as statistically significant predictors, while diastolic blood pressure (OR 0.98, 95% CI 0.96–1, *p* = 0.074) was not (model ROC AUC 0.745). When the model included only non-trauma patients (*n* = 161), it was confirmed that none of the IVC parameters were statistically significantly associated with massive transfusion (Supplemental Table 2).

**Table 5:** Logistic regression analyses of associations with massive transfusion

Parameter	Univariable OR	95% CI	*p*-value	Multivariable OR	95% CI	*p*-value
Age	0.99	0.97–1.02	0.517			
Sex (1 = male)	1.67	0.81–3.43	0.164			
Height	13.22	0.37–467.66	0.156			
Weight	1.02	1–1.04	**0.019**	1.03	1.01–1.05	**0.004**
BMI	1.06	1–1.13	**0.045**			
IVC volume, cm^3^	1.01	0.99–1.03	0.434			
IVC transv, diameter	0.97	0.9–1.04	0.397			
IVC AP diameter	1	0.95–1.06	0.962			
IVC flatness index	1.01	0.75–1.34	0.972			
SBP	0.99	0.98–1	0.268			
DBP	0.98	0.96–1	**0.029**	0.98	0.96–1	0.074
Heart rate	1	0.99–1.02	0.676			
SI	1.48	0.55–3.99	0.435			
DSI	1.36	0.89–2.08	0.16			
Hb level	0.56	0.41–0.76	**< 0.001**	0.54	0.39–0.74	**< 0.001**
PLT count	1	0.99–1	**0.041**			

OR, odds ratio; CI, confidence interval; BMI, body mass index; IVC, inferior vena cava; transv., transverse diameter; AP, anterior-posterior diameter; SBP, systolic blood pressure; DBP, diastolic blood pressure; SI, shock index (HR/SBP); DSI, diastolic shock index (HR/DBP); Hb, hemoglobin; PLT, platelet count; bold numbers indicate statistical significance (*p* < 0.05). Multivariable analysis including the three most significant predictors of body sizes, hemodynamic variables, and laboratory variables (providing 16.6 events per variable)

## Discussion

The present results suggest, that CT-derived IVC volume measurement provides different prognostic significance in a cohort of patients requiring transarterial embolization for acute bleeding. The IVC flatness index was significantly associated with 30-day mortality, but not with massive transfusion. IVC volumetry was not associated with either outcome.

Currently, there are no studies of similar patient cohorts with which to compare these results. A previous study from this center included 400 mechanically ventilated trauma patients from the scene and found that IVC volumetry was significantly associated with 24-hour mortality and blood transfusion. However, no significant associations were observed for 30-day mortality or massive transfusion [17].

Similar to the current findings, significant differences between the sexes were found in body height, body weight, and IVC volume [17]. Since men tend to be taller and heavier than women, this is reflected in IVC volume, which is larger in men. These results suggest that, although raw measurements of body size differ between the sexes, the calculated indices (BMI and IVC flatness index) normalize these differences, making them comparable. However, it must be acknowledged that BMI does not account for sex-related differences in body composition, particularly the variation in body fat percentage between men and women. Women typically have a higher percentage of body fat than men with the same BMI [18].

As previously mentioned, the assessment of IVC volume on imaging allows for the evaluation of the patient’s volume status and potential necessity for further interventions, such as fluid resuscitation and blood transfusion. A flattened IVC may be indicative of hypovolemia (low blood volume) and is associated with an increased risk of shock, major bleeding, blood transfusions, and mortality [19, 20].

With respect to the hemodynamic associations of the study results, diastolic blood pressure was the sole parameter that demonstrated a correlation with both IVC volume and IVC flatness index. From a physiological perspective, IVC volume is associated with right atrial pressure, thereby offering an indirect indication of diastolic blood pressure. A larger inferior vena cava (IVC) diameter and reduced collapsibility during respiration are associated with higher right atrial pressure and higher diastolic blood pressure [21]. Diastolic blood pressure has been identified as a significant prognostic indicator, particularly in patients with shock who require fluid resuscitation and vasopressors. Lower diastolic blood pressure levels have been shown to be indicative of reduced coronary perfusion and are associated with poorer outcomes. Conversely, elevated diastolic blood pressure, particularly in conjunction with heightened afterload, has been demonstrated to be a hallmark of diastolic dysfunction and a valuable indicator of heart failure [22].

The current patient cohort is marked by considerable heterogeneity, with a wide range of bleeding locations associated with varying mortality rates. This diversity may introduce a relevant bias, potentially leading to divergent conclusions. For example, it is understood that patients suffering from upper gastrointestinal bleeding have a poorer prognosis than those experiencing abdominal wall bleeding [23, 24].

It is important to note that the current mortality rate of 26.6% is relatively high, suggesting a high degree of case severity. In comparison, Powerski et al. reported a 30-day mortality of 18.4% in their cohort of 327 patients undergoing endovascular treatment [24]. The same study demonstrated that bleeding intensity and extent of injury were the only prognostic factors influencing clinical outcome [24]. However, the study did not evaluate the potential prognostic relevance of contrast extravasation and IVC volume.

Despite the high clinical and technical success of transarterial embolization, there is a lack of data on relevant prognostic factors in a cohort of patients with acute bleeding who are undergoing transarterial embolization. The present study contributes to the existing literature by suggesting that IVC-derived parameters can be considered a promising prognostic imaging marker. Further evaluation in this field is required in more homogeneous cohorts and with higher sample sizes. This will allow for better adjustment for potential confounding factors.

In a meta-analysis of 12 studies involving a total of 1,706 trauma patients, the diagnostic accuracy of flat IVC for predicting the development of shock was found to be acceptable (AUC 0.78), while its performance in predicting mortality was found to be suboptimal (AUC 0.60) [6]. A comprehensive review of the included studies showed significant differences in the methods used to measure IVC diameter at different levels. Furthermore, these studies employed IVC diameter ratios to determine IVC flatness, and volumetric analysis was not utilized.

A study of 236 trauma patients using IVC volumetry revealed a significant association between IVC volume and massive transfusion [14]. In this study, 18 patients (7.6%) underwent massive transfusion, which contrasts with the 90 patients (20.5%) in our previous study of trauma patients and the 50 patients (26.6%) in our present study [17]. Notably, both of these studies revealed no statistically significant association between IVC volume and massive transfusion.

The present study has some limitations to address. First, single-center retrospective studies are susceptible to potential biases. To mitigate potential bias, the imaging measurements were conducted in a blinded manner, independent of the clinical data. Second, the present analysis is subject to selection bias, as it only includes patients who underwent endovascular therapy. Third, the variety of underlying diseases was considerable, which makes it difficult to transfer as a representative cohort. Due to the heterogeneous study cohort presenting with various surgeries before and after the angiographic intervention (including surgeries in the same and other body regions), it is impossible to retrospectively determine complication and clinical success rates. Fourth, while the IVC volume measurement is a semiquantitative imaging analysis, it is not possible to rule out the possibility of investigator-related bias entirely. Additionally, the dataset revealed sex-specific IVC volumes, which must be considered in future studies. Fourth, a PRBC transfusion could introduce a confounding factor because it may be performed before or after the CT scan. This could influence IVC measurements. However, due to the retrospective design of the study, we could not adjust for this important factor. Similarly, we could not adjust for the administration of intravenous fluids, which could also confound the results.

## Conclusion

CT imaging-defined IVC flatness index but not IVC volume was significantly associated with 30-day mortality after adjustment for multiple predictors in patients with active bleeding undergoing endovascular therapy. Neither IVC volume nor flatness index transfusion was significantly associated with massive transfusion despite its previously identified association with overall volume status of the patients. Further validation studies with more homogeneous cohorts and higher sample sizes are required and to explore the potential prognostic value of IVC-derived parameters in critically ill patients.

## Electronic supplementary material

Below is the link to the electronic supplementary material.

Supplementary Material 1

## Data Availability

The datasets used and/or analyzed during the current study are available from the corresponding author on reasonable request.
