# Supplementary material for: The utility of computed tomography-derived inferior vena cava parameters in predicting outcomes in patients with active bleeding undergoing transarterial embolization
Source: Int J Emerg Med. 2025 Oct 20;18:211. doi: 10.1186/s12245-025-01033-9 (PMC12538849; doi:10.1186/s12245-025-01033-9)
Supplement: Supplementary file 1 — Supplementary Material 1 [file 12245_2025_1033_MOESM1_ESM.docx]

**Supplemental Table 1. Cox proportional hazard models of associations with 30-day mortality in non-trauma patients**

| **Parameter** | **Univariable**  **HR** | **95% CI** | **p-value** | **Multivariable**  **HR** | **95% CI** | **p-value** |
| --- | --- | --- | --- | --- | --- | --- |
| Age | 1.02 | 1 – 1.05 | 0.067 |  |  |  |
| IVC AP diameter | 0.93 | 0.88 – 0.99 | **0.016** |  |  |  |
| IVC flatness index | 1.45 | 1.16 – 1.8 | **0.001** | 1.38 | 1.10 – 1.75 | **0.006** |
| SBP | 0.99 | 0.98 – 0.99 | **0.014** |  |  |  |
| DBP | 0.97 | 0.96 – 0.99 | **0.003** |  |  |  |
| Heart rate | 1.02 | 1 – 1.03 | **0.016** |  |  |  |
| SI | 6.06 | 2.57 – 14.32 | **<0.001** | 4.95 | 2.11 – 11.65 | **<0.001** |
| DSI | 1.67 | 1.32 – 2.10 | **<0.001** |  |  |  |
| Hb level | 0.80 | 0.61 – 1.05 | 0.104 |  |  |  |
| pRBC (n) | 1.01 | 0.93 – 1.04 | 0.324 |  |  |  |
| Massive transfusion | 1.71 | 0.91 – 3.24 | 0.106 |  |  |  |

HR, hazard ratio; CI, confidence interval; IVC, inferior vena cava; AP, anterior-posterior diameter; SBP, systolic blood pressure; DBP, diastolic blood pressure; SI, shock index; DSI, diastolic shock index (HR/DBP); Hb, hemoglobin; pRBC, packed red blood cell units; bold numbers indicate statistical significance (p <0.05). Subgroup analysis of non-trauma patients (n = 161).

**Supplemental Table 2. Logistic regression analyses of associations with massive transfusion in non-trauma patients**

| **Parameter** | **Univariable**  **OR** | **95% CI** | **p-value** | **Multivariable**  **OR** | **95% CI** | **p-value** |
| --- | --- | --- | --- | --- | --- | --- |
| Age | 0.99 | 0.96 – 1.01 | 0.375 |  |  |  |
| Sex (1=male) | 1.55 | 0.71 – 3.39 | 0.265 |  |  |  |
| Height | 11.11 | 0.24 – 518.92 | 0.216 |  |  |  |
| Weight | 1.02 | 1 – 1.04 | **0.043** | 1.02 | 1.01 – 1.04 | **0.011** |
| BMI | 1.05 | 0.99 – 1.12 | 0.092 |  |  |  |
| IVC volume, cm^3^ | 1 | 0.97 – 1.03 | 0.923 |  |  |  |
| IVC transv, diameter | 0.96 | 0.89 – 1.04 | 0.35 |  |  |  |
| IVC AP diameter | 0.98 | 0.92 – 1.04 | 0.504 |  |  |  |
| IVC flatness index | 1.08 | 0.8 – 1.46 | 0.613 |  |  |  |
| SBP | 0.99 | 0.98 – 1 | 0.208 |  |  |  |
| DBP | 0.98 | 0.96 – 1 | 0.095 |  |  |  |
| Heart rate | 1 | 0.99 – 1.02 | 0.991 |  |  |  |
| SI | 1.42 | 0.48 – 4.18 | 0.53 |  |  |  |
| DSI | 1.23 | 0.78 – 1.95 | 0.373 |  |  |  |
| Hb level | 0.54 | 0.38 – 0.77 | **<0.001** | 0.50 | 0.34 – 0.72 | **<0.001** |
| PLT count | 1 | 0.99 – 1 | **0.026** |  |  |  |

OR, odds ratio; CI, confidence interval; BMI, body mass index; IVC, inferior vena cava; transv., transverse diameter; AP, anterior-posterior diameter; SBP, systolic blood pressure; DBP, diastolic blood pressure; SI, shock index (HR/SBP); DSI, diastolic shock index (HR/DBP); Hb, hemoglobin; PLT, platelet count; bold numbers indicate statistical significance (p <0.05). Subgroup analysis of non-trauma patients (n = 161).
